# Re-Vision of Geometric Flap Design in Lower Limb Reconstruction: A Comparative Analysis on Perforator Enhancement

**DOI:** 10.3390/jcm15020900

**Published:** 2026-01-22

**Authors:** Martino Guiotto, Daniel Schmauss, Giorgio Mariotti, Marco Morelli Coppola, Paolo Persichetti, Pietro Giovanni di Summa

**Affiliations:** 1 Department of Plastic, Reconstructive and Aesthetic Surgery, Ente Ospedaliero Cantonale, 6900 Lugano, Switzerland; 2 Faculty of Biomedical Sciences, University of Southern Switzerland (USI), 6900 Lugano, Switzerland; 3 Department of Plastic, Reconstructive and Hand Surgery, Centre Hospitalier Universitaire Vaudois (CHUV), 1011 Lausanne, Switzerland; 4 Department of Plastic, Reconstructive and Aesthetic Surgery, Fondazione Policlinico Universitario Campus Bio-Medico, 00128 Rome, Italy; m.morellicoppola@gmail.com (M.M.C.);

**Keywords:** local random flaps, perforator flaps, propeller flaps, lower limb reconstruction, flap geometry, length width ratio, flap design

## Abstract

**Background:** Perforator flaps improved the reconstruction paradigm in the lower extremity, increasing coverage possibilities. This study aims to quantify how added perforators could enhance standard geometrical patterns (compared to random flaps). **Methods:** A total of 29 cases of lower limb soft tissue reconstruction (STR)—52% trauma, 21% osteomyelitis with skin fistulas, 21% healing disorders with unstable scarring and 6% cancer-related surgery—were performed in our institution between 2012 to 2023 with geometric random (GR) local flaps (34%), geometric perforator-enhanced (GP) flaps (32%) or pure propeller perforator (PP) flaps (34%), were retrospectively analysed. Patients with proximal thigh defects, a follow up of less than 3 months and those who received an axial, muscle or free flap were excluded. Geometric patterns (as length:width ratio (L:W)) were compared among groups, analysing healing outcomes and complications. **Results:** Leg defects were categorized into 62% distal, 14% middle, 14% proximal third and 10% distal thigh. No significant difference in defect size was detected among groups. Mean flap size was significantly larger for GP (70.5 cm^2^, (*p* < 0.05)) and PP (74.4 cm^2^, (*p* < 0.01)) than GR (53.7 cm^2^). The L:W ratio was significantly higher in GP (L:W 2.2:1, (*p* < 0.05)) and PP (L:W 2.8:1, (*p* < 0.01)) than in GR (1.5:1), but no significant difference was found between GP and PP. A reduced complications rate (partial flap loss, infection, healing, revision surgery, etc.) was observed in the GP group, when compared to GR. **Conclusions:** Flap geometric design can be significantly improved by the inclusion of perforators, maintaining spatial advantages with larger ductility and improved vascular solidity.

## 1. Introduction

Lower extremity defects are frequently secondary to trauma, infection, wound healing disorder or cancer-related surgery [1,2]. Defect coverage can be complicated by potentially diminished vascularity or jeopardization of the leg and minimal skin laxity, making the reconstructive process rapidly climb up the reconstructive ladder [3]. Indeed, considering the thin, non-expandable soft tissue coverage of the lower limb, even smaller defects can result in a challenging reconstructive scenario, especially in the presence of bone, tendon or hardware exposure [4,5].

Traditionally, the reconstructive ladder for complex lower extremity defects consisted of regional pedicled muscle flaps for the thigh and the proximal and middle third of the leg, while free flaps are mainly indicated for the distal third of the leg and foot [6,7,8]. Local skin flaps used to be relegated to smaller defects due to the random subdermal vascularization, which traditionally limits the length-to-width (L:W) ratio between 1:1 and 1.5:1 [9,10,11]. Additionally, local flaps can be further characterized regarding their geometric pattern by their primary harvesting movement: advancement, rotation and transposition. In the first type, the main flap movement is along a linear direction toward the primary defect. Commonly, an advancement flap has a L:W ratio between 1:1 or 2:1, with a significant variation between different anatomical sites [12].

Rotation flaps are characterized by a rotation (30° or less) along an arc around a fixed pivot point at the base of the flap [13].

Flaps are raised from an adjacent donor site and moved into the defect. Some examples of transposition flaps are the rhomboid (Dufourmentel and Limberg) or bilobed flap [14,15,16].

Local fasciocutaneous perforator flaps and propeller flaps can effectively address larger defects while significantly reducing donor site morbidity [4,17]. This makes them a valuable alternative to free flaps, especially for reconstruction of middle and distal third lower limb defects [18]. The inclusion of at least one perforator in the flap enhances vascular reliability, allowing for greater flap dimensions and increased advancement, rotational or transposition movement, thereby redefining traditional geometric constraints.

However, the geometric design limitations of perforator flaps have yet to be fully delineated in the current literature. By analysing flap-related and wound-healing complications, along with revision surgery rates, the present study aims to evaluate the extent to which traditional geometric design principles may be modified following the inclusion of perforators.

## 2. Materials and Methods

A retrospective study was conducted on a cohort of 29 patients who underwent a local flap-based reconstruction of the lower limb at Lausanne University Hospital between 2012 and 2023. This study was conducted in accordance with the ethical standards of the institutional and national research committee and with the 1964 Helsinki Declaration. This study received the approval from the local ethics committee (CER-VD 2024-00178). All patients signed a written consent.

Out of approximately 50 lower limb reconstructions performed per year in the institution and recorded in a prospectively maintained retrospective database, inclusion criteria were applied. They consist of adult patients who underwent soft tissue reconstruction (STR) for distal thigh, proximal (or knee), middle and distal third leg defects (including dorsal foot), exclusively with a random local flap or perforator flaps. Free flaps, muscle local flaps and limb reconstructions of the proximal thigh were excluded, as well as patients who did not complete a minimum of 3 months follow-up.

### 2.1. Surgical Timing

STR was generally planned as early as possible. In patients who underwent a one-stage procedure, the STR was performed directly after debridement, antibiotics and framework material exchange, when present. Cases were discussed in a multi-disciplinary board (infectologists, oncologists and orthopaedic and plastic surgeons).

### 2.2. Outcome Analysis

Defect size, flap type, dimensions and eventual rotation arch were recorded. Length-to-width ratio (L:W) was calculated in order to define and quantify the geometrical enhancement of perforator flaps compared to the random local flaps. Based on the operative notes, flap length and base width were recorded using intraoperative measurements obtained with a ruler. Flap length was defined as the maximum longitudinal dimension of the flap, while flap width corresponded to the width of the flap base. The L:W ratio was calculated using the formula r = L/W and expressed as a proportion to 1 (L/W:1), in accordance with the majority of the published literature. For each flap category, the average L:W ratio was calculated as the mean of the individual L:W ratios.

Healing outcomes and post-operative complications (hematoma, seroma, infection (including recurrent osteomyelitis) and delayed wound healing (defined as more than 3 weeks)) were recorded. Besides that, flap complications (total and partial flap loss, this last defined as any partial flap necrosis requiring a supplementary revisional surgery [19]) as well as surgical time, hospitalization length and mobility day were collected.

### 2.3. Surgical Technique

1. Random flaps

Random local flaps were skin and subcutaneous tissue harvested according to the most common geometrical patterns, such as advancement, rotation and transposition. Once the flap was harvested, it was shifted into the defect without tension. The donor site was closed directly or skin grafted.

2. Perforator flaps

Acoustic Doppler ultrasound was used for preoperatively localizing reasonable perforators. Provisional design was then defined according to the defect characteristics and flap chosen. When designing a propeller flap, the axis of the flap was oriented in a longitudinal direction parallel to the long bones of the leg, in order to capture adjacent perforasomes [20].

Approach to perforators was subfascial. Once visualized, the perforators were tested in terms of size and pulsatility. Intramuscular dissection of the chosen perforator was performed (if needed) to reduce the risk of perforator kinking or torsion after flap mobilization and setting. The skin incision around the skin paddle was then completed. The flap was harvested and rotated/translated into the defect. The donor site was closed directly or skin grafted.

### 2.4. Postoperative Rehabilitation and Physiotherapy Protocols

Patients followed a postoperative protocol of 3 days in bed with limb elevation and without cast immobilization. Orthostatic position was progressively achieved from day 3 to 7, associated with class II compression garments.

### 2.5. Statistical Analysis

All data were expressed as average ± standard error (SE) of the mean. Comparison between groups was performed with parametric or nonparametric statistical tests (one-way ANOVA multiple comparison test, student *t* test or Mann–Whitney) when appropriate. To verify the data spread, the D’Agostino and Pearson omnibus normality test was applied.

Significance was expressed as * *p* < 0.05, ** *p* < 0.01, *** *p* < 0.001 and **** *p* < 0.0001. All analysis was performed using GraphPad Prism 8 (GraphPad Software, La Jolla, CA, USA).

## 3. Results

The patient’s demographic data, flaps design and flap-related outcomes were outlined in the Table S1. The study included 29 patients (14 females, 48.3%) with a mean age of 63 years.

According to the aetiology, 52% needed STR for trauma, 21% for infection/osteomyelitis, 21% for chronic wounds and 6% due to an oncological resection.

In terms of anatomical defect distribution, 62% of the defects were localized at the distal third (ankle and foot included), 14% the middle third and 14% the proximal third (knee included); 10% were thigh defects.

According to the surgical technique, GR was used in 34% of patients, while 32% of patients received GP; in 34% a PP was harvested.

Defect size following the debridement was 26.6 cm^2^ on average (range 3–150 cm^2^), while the mean size of the flap was 66.1 cm^2^ (range 10–300 cm^2^).

The donor site was closed primarily in 16.1% of the cases, while in the remaining 83.9% a skin graft was performed.

The mean follow-up in our study was 15 months (range 4–61).

### 3.1. Geometric Random Local Flaps (GR)

Defect size was in mean 31.7 ± 4.7 (SEM) cm^2^. The average size of the harvested flap was 53.7 ± 4.4 (SEM) cm^2^. Mean L:W ratio resulted in 1.5 ± 0.1 (SEM):1. The flap type was advancement in 3 out of 10, rotational in 4 out of 10, transposition in 3 out of 10 (2 out of 3, Dufourmentel).

No full flap loss was recorded among the complications. Among the other complications, partial flap loss, delayed wound healing and osteomyelitis each occurred in 20% of cases. The revision surgery rate was 30% with a mean hospitalization time of 19 days.

### 3.2. Geometric Perforator—Enhanced Flaps (GP)

Mean defect size was 19.2 ± 6.1 (SEM) cm^2^. The average flap size was 70.5 ± 16.8 (SEM) cm^2^. L:W ratio resulted in average of 2.2 ± 0.3 (SEM):1. The flap type was rotation (2 out of 9) or transposition (7 out of 9 including Dufourmentel (1) and Hatchet flap (2)).

Among complications, we recorded 0% total flap loss, 11.1% partial flap loss, 0% delayed wound healing, 11.1% osteomyelitis and 11.1% revision surgery, with a mean hospitalization time of 9 days.

### 3.3. Pure Perforator Propeller Flaps (PP)

Mean defect size was 28.1 ± 5.9 (SEM) cm^2^. Mean flap size was 74.4 ± 9.9 (SEM) cm^2^. L:W ratio was 2.8 ± 0.2 (SEM):1. The flap was an island propeller perforator (in all cases) based on lateral supra genicular artery perforator (3 out of 10), posterior tibialis artery perforator (5 out of 10) or peroneal artery perforator (2 out of 10).

Among complications, we recorded 20% total flap loss, 20% partial flap loss, 30% delayed wound healing, 30% osteomyelitis and 50% revision surgery, with a mean hospitalization time of 24 days.

**Figure 1:**
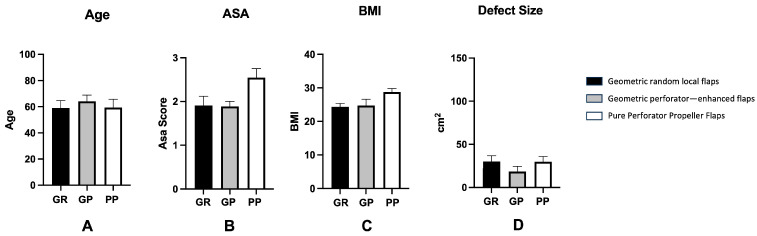
This graph shows demographic and clinical data (Age, Sex, BMI and Defect Size) in the three groups. No significant difference among the groups was found in each of these categories. All data were expressed as average ± standard error (SE) of the mean. A one-way ANOVA multiple comparison test was performed.

Overall, no significant difference was found in patient age, sex distribution, BMI, ASA score and defect size among the experimental groups (Figure 1).

**Figure 2:**
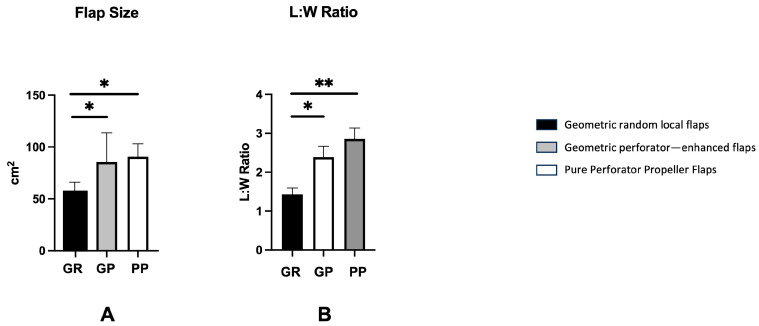
In this graph, the three techniques are compared in terms of size of flap (**A**) and the length:width ratio (**B**). The flap size and L:W ratio were significantly higher in the enhanced perforator and pure propeller perforator flaps when compared to random flaps. No significant difference was found among enhanced perforator and pure propeller perforator flaps. All data were expressed as average ± standard error (SE) of the mean. One-way ANOVA multiple comparison test was performed. Significance was expressed as * *p* < 0.05, ** *p* < 0.01.

The flap size was significantly larger for GP (*p* < 0.05) and PP (*p* < 0.01), when compared to the GR (GP: 70.5 cm^2^ vs. PP: 74.4 cm^2^ vs. GR: 53.7 cm^2^) (Figure 2A). The L:W ratio was significantly higher in GP (*p* < 0.05) and PP (*p* < 0.01) than in GR group. (GP: 2.2 vs. PP: 2.8 vs. GR: 1.5). However, no significant difference was found between GP and PP in terms of L:W ratio (Figure 2B). In the GR group, all patients received a skin graft, 55% of the patients received it in GP group (75% in PP).

**Figure 3:**
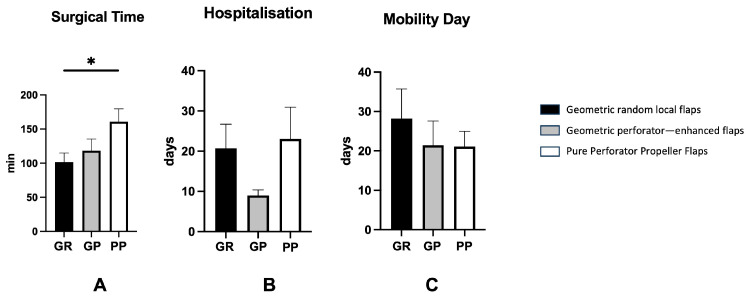
This graph shows the three different techniques compared by surgical time (**A**), hospitalization length (**B**) and recovering mobility (**C**). The surgical time was significantly longer in the pure propeller perforator flaps when compared to the random flaps. The hospitalization length and mobility day did not show any significant difference between three groups. All data were expressed as average ± standard error (SE) of the mean. One-way ANOVA multiple comparison test was performed. Significance was expressed as * *p* < 0.05.

Moreover, surgical time was significantly elevated in the PP group when compared to GR (*p* < 0.05), but not significant between GR and GP or GP and PP (GP: 118 min vs. PP: 147 min vs. GR: 103 min) (Figure 3A). No significant difference in hospitalization time was recorded between GR, GP and PP (Figure 3B). Finally, no significant difference in time until mobilisation was recorded between the groups (Figure 3C).

To summarise, GP and PP offered a significant more extensive coverage than GR. However, while PP showed a higher complication rate among the groups, GP impacted less in terms of flap loss, delayed wound healing, infection, and revision surgery even when compared with random flaps.

## 4. Discussion

Lower limb defects are traditionally reconstructed using STSG, random flaps, regional pedicled muscle flaps or free tissue transfer [21]. STSG are a simple option, particularly when bone and neurovascular bundles are not exposed; however, poor cosmesis due to scar retraction limits their applicability. On the contrary, flaps are considered a preferred option in case of bone or tendon exposure [22]. Random flaps can provide a satisfactory soft tissue coverage, but their applicability in the lower extremity is limited by a low L:W ratio [23,24,25].

In the last two decades, reconstructive algorithms have been revolutionized by perforator flaps: these have enlarged indications, types and number of available local flaps [26]. Moreover, the lower extremity is the largest donor site for perforator flaps: an anatomical study by Morris et al. showed 93 perforators from 21 territories with relatively constant perforators from the 3 major vessels, allowing predictable and reliable flap harvesting [27].

Indeed, the interest around perforator flaps lead surgeons to re-think the traditional geometry in an attempt to offer a targeted and like-to-like reconstruction for larger defects [26,28]. As result, the prediction of the safe dimensions of perforator flaps is paramount in pre-surgical planning and flap design.

The current study investigated the relation between the L:W ratio for GR, GP and PP flaps in lower limb reconstruction. Healing outcomes, complications and eventual revision surgeries were assessed for patients treated with the techniques mentioned above. It aimed to demonstrate if and how perforator flaps can enhance the L:W ratio and generally extend the traditional geometric design of GR. Second, it defined a balanced L:W proportion, in order to limit wound healing complications and maximize the coverage with perforator flaps.

**Figure 4:**
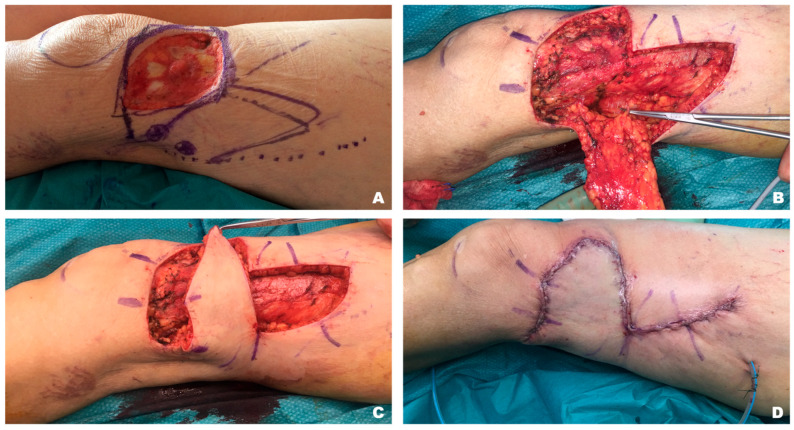
Distally based enhanced perforator flap for a distal thigh defect coverage. Defect pre debridement and preoperative drawings (**A**), flap harvesting and perforators identification (**B**), flap mobilisation and inset (**C**), defect closure and final scars (**D**).

**Figure 5:**
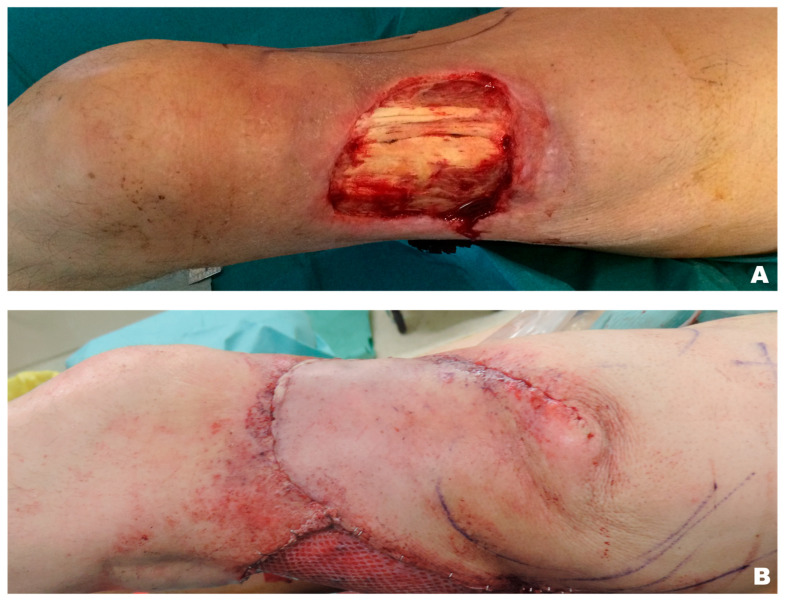
Proximally based random flap for a distal thigh defect coverage with skin grafted donor site. Defect pre debridement (**A**), flap inset and closure (**B**).

When considering GR and perforators flaps, the flap size resulted significantly increased with a higher L:W ratio for perforator flaps. Particularly, a L:W ratio of 2.8:1 could be achieved with propellers (PP), whereas a L:W ratio at 2.2:1 was recorded using “extended” geometric design in GP, compared with a L:W ratio at 1.5:1 of the random flaps (GR) (*p* < 0.01 and *p* < 0.05) (Figure 4 and Figure 5).

In terms of healing outcomes, a meaningful reduced complications rate in GP group was observed, when compared to GR, in terms of partial flap loss, infection, delayed healing and revision surgery. When PP are considered, a significant increased incidence of total flap loss, partial flap loss and revision surgery were registered when compared to GR and GP groups.

**Figure 6:**
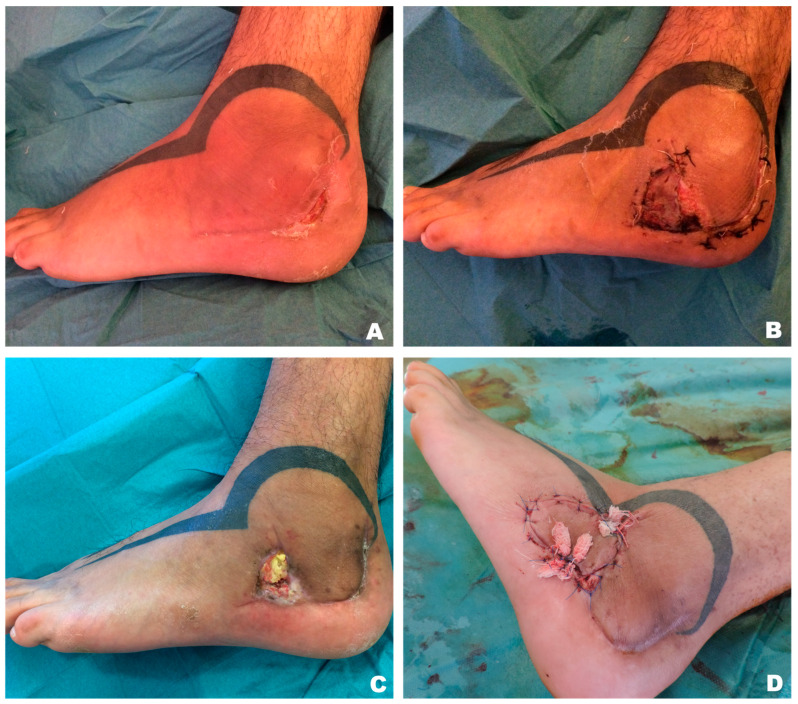
Cutaneous bony fistula at lateral malleolus (**A**) covered initially by a transpositional random flap and skin graft at the donor site (**B**). Following a partial flap and total skin graft loss (**C**), an island enhanced perforator flap was performed to close successfully the defect (**D**).

Indeed, enhanced geometric perforator flaps seemed to be a safe procedure even with increased flap dimensions, allowing wider coverage than traditional random flaps, but keeping the complication rates lower compared to propellers (Figure 6).

Initially, the use of perforator flaps in lower limb reconstruction was described by Hallock, who included the deep fascia within local skin flaps. This allowed him to avoid free flaps, particularly in ankle and lower third leg defects, offering a reliable and quicker inset with an overall complication rate of 19% (partial or total flap necrosis). Of 38 patients, 36 reported satisfactory reconstruction without further surgeries [10].

Besides that, Hallock recommended a longitudinal axis-oriented flap along an intermuscular septum. In the author’s experience, the inclusion of fascial perforators was more relevant than flap width, which should be limited to an L:W ratio of 1.8:1 to ensure total flap survival [11].

In line with this study, Cheng et al. reported their experience with peroneal artery perforator flaps for chronic extremity wound coverage. The average operating time was 132 min, and flap sizes ranged between 1.5 and 260 cm^2^. All 55 flaps survived; however, partial flap loss occurred in 18.2% of cases, necessitating revision surgery with debridement followed by STSG. The average hospital stay was 33.4 days. All patients reported satisfaction with the functional and cosmetic outcome [29]. In their series, the mean L:W ratio was 2:1, comparable to our 2.2:1 L:W ratio for the enhanced geometric perforator group.

On the one hand, when considering random skin flaps, despite the limited coverage, the reliability and low complication rate have been confirmed by multiple studies, such as Uyar et al. The authors analysed the outcomes of 23 patients with lower limb defects (mean size 7.6 cm^2^) covered by a random bipedicle flap. Infection was reported in 5 cases and wound dehiscence in 3, but all were managed conservatively [30]. Compared to our study, the mean defect size was significantly lower (7.6 vs. 31.7 cm^2^): this might explain the higher complication rate in our patients, particularly in terms of partial flap loss, delayed wound healing and revision surgery.

On the other hand, regarding perforator propellers flaps, our findings are in line with previous studies, confirming the increased risk of wound healing complications and flap loss, mainly related to an eventual perforator kinking during flap rotation (excessive rotation angle) and inset, excessive flap length and suboptimal perforator selection or venous congestion as well as tension at closure, compromised or inflamed recipient tissue and patient-related factors [31,32,33]. The most common complications are partial flap loss (11%), limited or reversible venous congestion (8%), epidermolysis (4%) and revision surgery (6.5%) [28,34].

Moreover, Panse et al. investigated healing outcomes of propeller flaps in relation to the maximal distance of the flap from its perforator. They referred this distance to the limb length, measured from the superolateral aspect of the lateral malleolus to the fibular head. Overall, they found flap necrosis six times more frequent if the distance between the flap distal edge to its perforator is longer than one-third of the limb length. Thus, propellers should to be designed with a maximum length equal to one-third of limb length [26].

Similarly, the average L:W ratio in our study was 2.8:1, a value that approximates the one-third limb length ratio reported by Panse et al., While the rate of partial flap loss was comparable between the two studies (20% vs. 20%), the incidence of total flap loss was significantly higher in our cohort (20% vs. 8.6%). Interestingly, total flap loss occurred with L:W ratios of both 4:1 and 2.3:1, respectively. This contrasts with the findings of Panse et al. described above. Unfortunately, the limited number of patients in both studies constrains further data interpretation.

Lastly, differently from propeller designs, some defects may be successfully covered by geometric design based on multiple perforators around the defect to maximize arterial and venous flap perfusion [35]. Typically, when harvested as multi-perforator fasciocutaneous advancement flap, keystone geometric design can be centred over a dominant perforator, allowing more extensive undermining and the raising of a wider flap compared to the traditional keystone geometric pattern, depending on regional tissue laxity and vascularization [36]. The same concept can be applied to a V–Y design flap, based on individualized perforators, which shows an improved arterial flow and a decreased venous congestion risk [37,38].

Overall, the current study reinforces the evolving role of perforator flaps in lower limb reconstruction, particularly in expanding the limitations imposed by traditional random flap geometry. Enhanced geometric perforator flaps demonstrated a favourable balance between increased flap dimensions and reduced complication rates, offering a reliable and safe alternative. While propeller flaps allow for an even greater L:W ratio, their higher complication rates warrant cautious application and meticulous planning.

Ultimately, careful flap design based on individualized perforator mapping and respecting anatomical constraints such as limb length proportions appears essential for optimizing outcomes.

### Limitations

The small sample size as well as the retrospective nature of our study might lead to unavoidable bias in outcome interpretation. In addition, lack of comparison between the island random geometric keystone flaps and the island geometric perforator flaps represent a further limitation due to the unpredictable and non-comparable skin bridge effect on flap vascularization. In our centre, we usually perform free flap surgery when frameworks are exposed in lower limb reconstruction [34]; indeed, the number of patients included in this study was limited. Finally, we excluded muscle pedicled and sural flaps, further reducing the patient selection.

However, patient characteristics and defect types among the three groups were homogeneously distributed.

## 5. Conclusions

This work aimed to understand if and how perforator flaps can enhance random flap design in lower limb reconstruction. Enhanced geometry perforator flaps allowed coverage of wider defects than traditional random flaps, keeping the overall complication rate significantly low.

While choices towards microsurgery are increasing, re-embracing local flaps, particularly with the enriched armamentarium of perforator flaps, can offer an additional valuable tool in soft tissue reconstruction. Even, when a random flap can be considered, a perforator should always be included, aiming for an extended geometric flap design, assuring a safer, reliable, and more effective coverage.

## Data Availability

The original contributions presented in this study are included in the article/Supplementary Materials. Further inquiries can be directed to the corresponding author(s).

## Supplementary Materials

The following supporting information can be downloaded at: https://www.mdpi.com/article/10.3390/jcm15020900/s1, Table S1: Patient demographics, clinical data, flap design and flap-related outcomes. We indicated by “mean” the average value for each group (GR, GP and PP) in each column, when applicable. The “overall mean” represents the average value for each column, when applicable, calculated across all patients, regardless of flap type.

## Abbreviations

The following abbreviations are used in this manuscript:

STR	soft tissue reconstruction
GR	geometric random local flaps
GP	geometric perforator—enhanced flaps
PP	pure propeller perforator flaps
L:W	length to width ratio
SE	standard error
STSG	split thickness skin graft

## References

[1] Soltanian H., Garcia R.M., Hollenbeck S.T. (2015). Current Concepts in Lower Extremity Reconstruction. Plast. Reconstr. Surg..

[2] AlMugaren F.M., Pak C.J., Suh H.P., Hong J.P. (2020). Best Local Flaps for Lower Extremity Reconstruction. Plast. Reconstr. Surg. Glob. Open.

[3] Sapino G., Osinga R., Maruccia M., Guiotto M., Clauss M., Borens O., Guillier D., di Summa P.G. (2023). Role of the Gastrocnemius Musculocutaneous with a Propeller Style Skin Flap in Knee Region Reconstruction: Indications and Pitfalls. Arch. Plast. Surg..

[4] Hallock G.G. (2013). A paradigm shift in flap selection protocols for zones of the lower extremity using perforator flaps. J. Reconstr. Microsurg..

[5] Lee Y.J., Park M.C., Park D.H., Hahn H.M., Kim S.M., Lee I.J. (2017). Effectiveness of Acellular Dermal Matrix on Autologous Split-Thickness Skin Graft in Treatment of Deep Tissue Defect: Esthetic Subjective and Objective Evaluation. Aesthetic Plast. Surg..

[6] Innocenti M., Menichini G., Baldrighi C., Delcroix L., Vignini L., Tos P. (2014). Are there risk factors for complications of perforator-based propeller flaps for lower-extremity reconstruction?. Clin. Orthop. Relat. Res..

[7] Bekara F., Herlin C., Mojallal A., Sinna R., Ayestaray B., Letois F., Chavoin J.P., Garrido I., Grolleau J.L., Chaput B. (2016). A Systematic Review and Meta-Analysis of Perforator-Pedicled Propeller Flaps in Lower Extremity Defects: Identification of Risk Factors for Complications. Plast. Reconstr. Surg..

[8] Martineau J., Guillier D., Maruccia M., Guiotto M., Borens O., Raffoul W., di Summa P.G. (2022). Locoregional anesthesia for post-operative pain management in microsurgical reconstruction of the lower extremities: A retrospective study. J. Plast. Reconstr. Aesthet. Surg..

[9] Maciel-Miranda A., Morris S.F., Hallock G.G. (2013). Local flaps, including pedicled perforator flaps: Anatomy, technique, and applications. Plast. Reconstr. Surg..

[10] Hallock G.G. (1989). Local fasciocutaneous flaps for cutaneous coverage of lower extremity wounds. J. Trauma..

[11] Hallock G.G. (1990). Distal lower leg local random fasciocutaneous flaps. Plast. Reconstr. Surg..

[12] Limberg A.A. (1946). Mathematical Principles of Local Plastic Procedures of the Surface of the Human Body.

[13] Bar-dach J. (1992). Local Flaps and Free Skin Grafts in Head and Neck Reconstruction.

[14] Larrabee W.F. (1990). Design of local skin flaps. Otolaryngol. Clin. N. Am..

[15] Murakami C.S., Nishioka G.J. (1996). Essential concepts in the design of local skin flaps. Facial Plast. Surg. Clin. N. Am..

[16] Patel K.G., Sykes J.M. (2011). Concepts in local flap design and classification. Oper. Tech. Otolaryngol..

[17] Guiotto M., di Summa P.G., Argentino G., Cherubino M. (2021). The proximal ulnar perforator flap (PUPF) for hand digital reconstruction: An anatomical study. J. Plast. Surg. Hand Surg..

[18] Mohan A.T., Sur Y.J., Zhu L., Morsy M., Wu P.S., Moran S.L., Mardini S., Saint-Cyr M. (2016). The Concepts of Propeller, Perforator, Keystone, and Other Local Flaps and Their Role in the Evolution of Reconstruction. Plast. Reconstr. Surg..

[19] Guillier D., Sapino G., Schaffer C., Borens O., Thein E., Bramhall R., di Summa P. (2021). Lower limb reconstruction involving osteosynthesis material: A retrospective study on propeller flaps outcomes. Injury.

[20] Saint-Cyr M., Wong C., Schaverien M., Mojallal A., Rohrich R.J. (2009). The perforasome theory: Vascular anatomy and clinical implications. Plast. Reconstr. Surg..

[21] Rad A.N., Singh N.K., Rosson G.D. (2008). Peroneal artery perforator-based propeller flap reconstruction of the lateral distal lower extremity after tumor extirpation: Case report and literature review. Microsurgery.

[22] Dini M., Innocenti A., Russo G.L., Agostini V. (2001). The use of the V-Y fasciocutaneous island advancement flap in reconstructing postsurgical defects of the leg. Dermatol. Surg..

[23] Ahn D.K., Lew D.H., Roh T.S., Lee W.J. (2015). Reconstruction of Ankle and Heel Defects with Peroneal Artery Perforator-Based Pedicled Flaps. Arch. Plast. Surg..

[24] di Summa P.G., Sapino G., Cherubino M., De Santis G., Durand S., Zaugg P., Bauquis O., Raffoul W. (2019). Reconstruction of complex soft tissue defects including tendons with anterolateral thigh flap extended to fascia lata: Long term recovery and functional outcomes. Microsurgery.

[25] Sapino G., Zaugg P., Cherix S., Borens O., Lo S., Raffoul W., di Summa P. (2019). ALT flap with vascularized fascia lata for one-stage functional patellar tendon reconstruction. J. Plast. Reconstr. Aesthet. Surg..

[26] Panse N.S., Bhatt Y.C., Tandale M.S. (2011). What is safe limit of the perforator flap in lower extremity reconstruction? Do we have answers yet?. Plast. Surg. Int..

[27] Morris S.F., Tang M., Almutari K., Geddes C., Yang D. (2010). The anatomic basis of perforator flaps. Clin. Plast. Surg..

[28] Gir P., Cheng A., Oni G., Mojallal A., Saint-Cyr M. (2012). Pedicled-perforator (propeller) flaps in lower extremity defects: A systematic review. J. Reconstr. Microsurg..

[29] Cheng L., Yang X., Chen T., Li Z. (2017). Peroneal artery perforator flap for the treatment of chronic lower extremity wounds. J. Orthop. Surg. Res..

[30] Uyar I., Aksam E., Yit K. (2023). Reconstruction option in complex lower extremity defects where microsurgical repair is not possible: Randomized bipedicled flaps. Ulus. Travma Acil Cerrahi Derg..

[31] Blough J.T., Saint-Cyr M.H. (2021). Propeller Flaps in Lower Extremity Reconstruction. Clin. Plast. Surg..

[32] Song P., Pu L.L. (2021). Perforator-Plus Flaps in Lower Extremity Reconstruction. Clin. Plast. Surg..

[33] Huang J., Yu N., Long X., Wang X. (2017). A systematic review of the keystone design perforator island flap in lower extremity defects. Medicine.

[34] di Summa P.G., Sapino G., Wagner D., Maruccia M., Guillier D., Burger H. (2023). Combined Free Flaps for Optimal Orthoplastic Lower Limb Reconstruction: A Retrospective Series. Medicina.

[35] Pripotnev S., White C. (2017). The Keystone Flap-A Case Series Demonstrating Practical Design, Use, and Applications. Plast. Surg..

[36] Monarca C., Rizzo M.I., Sanese G. (2012). Keystone flap: Freestyle technique to enhance the mobility of the flap [corrected]. ANZ J. Surg..

[37] Kerfant N., Monnerie C., Henry A.-S., Ta P., Hu W., Letissier H., Le Nen D. (2018). Posterior tibial perforator-based flaps for leg and foot defects: Indications, limitations, and technical considerations. Orthop. Traumatol. Surg. Res..

[38] Almishal O., Bin Mahmoud A.Z., Alshomer F.M. (2022). Posterior Tibial Artery Perforator V-Y Advancement Flap for Small-Medium Size Lower Limb Defects. Plast. Reconstr. Surg. Glob. Open.

